# Compliance to “Unpleasant” actions of crisis management: some remarks from a management control perspective

**DOI:** 10.1007/s11299-020-00250-4

**Published:** 2020-08-06

**Authors:** Friederike Wall

**Affiliations:** Department of Management Control and Strategic Management, University of Klagenfurt, Universitätsstraße 65-67, 9020 Klagenfurt, Austria

**Keywords:** Agent-based modelling, Covid-19, Management control, Social approval, Risk perception, Voluntary cooperation

## Abstract

In managing the Covid-16 pandemic, policy makers took actions which require the cooperation of individual citizens to succeed while the actions partially come at remarkable costs for individuals. The brief paper employs a thought experiment to identify factors which affect individuals’ propensity to cooperate in the public goods game. These factors reasonably comprise, for example, risk perception and attitude towards risk, embeddedness in a social network or the desire for social approval and may differ remarkably among the individuals of a collective. The paper adopts a management control perspective which appears to be particularly helpful to identify how to implement policy makers’ actions with respect to the diverse individuals in a collective. In order to predict the overall outcome of “unpleasant” actions, an approach is required which allows to capture the heterogeneity of individuals within a collective which makes agent-based modelling a promising candidate.

## Introduction

In the Covid-19 pandemic a lot of actions are taken by policy makers to inhibit the further spread of the pandemic. These actions comprise “lock-downs” including not only, for example, universities and schools but also interventions in the personal freedom of people. These interventions are of a kind that was, so far, unseen in many countries and include limitations of free movement or meeting with family and friends. The consequences for most of us are, at least, “unpleasant”. At the same time, in many countries, the enforcement of these actions is not so strict that there would be absolutely no decision-making scope which means that to some extent individuals also face situations of voluntary cooperation (Holländer 1990). The private so-called “Corona parties” may serve as an example of non-cooperative behaviour in the Covid-19 crisis.

However, the apparent compliance of a vast majority of people in Europe may be a worthwhile subject of research. In this short note, I take a “management control” perspective and—starting with some kind of “thought experiment” —the attempt is made to figure out some lines for future research related to the linkage of micro-level and macro-level perspective on compliance to “unpleasant” actions taken by authorities for crisis management.

## A thought experiment from a management control perspective

Management control could be regarded as a sub-domain in managerial science and is concerned with questions of how to align the behaviour of employees and, in particular, the decisions taken by managers with the overall objectives of an organization (e.g., Merchant and Van der Stede 2017). For this, management control provides a multitude of devices and techniques—so called “controls”. Following Merchant and Van der Stede (2017), these are results controls (e.g., incentive schemes), action controls (setting behavioural constraints), personnel controls (e.g., selection and placement of employees) and cultural controls (e.g., norms and beliefs). For the prominent framework of Levers of Control (LOC) see Simons (1994); Widener (2007).

Given the long-standing tradition of economics and, in particular, principal-agent theory with the respective analytical approaches in the domain of management control (Guffey and Harp 2017), it is a worthwhile attempt to rely on this perspective for a start.

In the thought experiment, the overall objective related to the spread of the pandemic may come along as “flattening the curve” or (reduction of) the reproduction rate. The problem of the authorities is, hence, to align the single individuals’ behaviour to the overall objective. For the sake of the argument in the experiment, now I think of an individual A as a young adult person in good shape, i.e., not part of the risk group of Covid-19. In face of the aforementioned “unpleasant” actions and the level of enforcement taken by the authorities, individual A has to decide (implicitly or explicitly) about the level of compliance to (or cooperation in) the authorities’ unpleasant actions.

From an economic perspective the next step is to ask for the utility function of individual A and the particular components of individual A’s utility function which may be relevant in that situation. Reasonably, at least, the following components could be relevant:

1. A’s risk to get infected by the pandemic which decreases with a higher level of compliance; this accounts for A’s *expected level of health*.
2. A’s level of compliance contributes to the overall objective (“flattening”) and, thus, shortens the *expected remaining duration* of the unpleasant actions and their *expected further consequences* on A’s financial wealth, A’s social distancing from family and friends etc.
3. Should A not cooperate, i.e., not comply to the “unpleasant” actions, there is the risk of punishment where the *expected level of punishment* from the executive authorities is shaped by A’s level of compliance and by the probability that an eventual uncooperative behaviour is uncovered and prosecuted.
4. A’s level of cooperation is also relevant with respect to social or moral norms, i.e., (not) violating norms and (not) getting ashamed accordingly which refers to *expected social approval*.

These four components are not meant to be complete, they just serve for the thought experiment which continues as follows. According to the behavioural assumptions of traditional schools of economic thought, our individual A is able to assess all the expected values without systematic errors.

With individual A being a young adult in good shape, the aforementioned component 1 would be fairly negligible. Regarding component 2—i.e., A’s individual contribution to the overall objective—one may argue that this depends on the structure of the social network in which A is residing in and A’s position in the network. However, regarding the situation as a public good game, the own contribution to the overall objective (i.e., “flattening the curve” of an entire country) is vague and presumably negligible which, per se, suggests a rather low propensity for cooperation (Holländer 1990; Kölle 2015). The third component addresses consequences related to enforcement and punishment taken by the authorities and refers to what in management control is called “action controls”. In tendency, the higher the punishments the higher the *cost of uncooperative behaviour* for individual A. The fourth component refers to “cultural controls” and the social approval desired by individual A. This also refers to Holländer (1990) who proposes a model providing an economic understanding of voluntary cooperation as social exchange and relates it to governmental interventions.

Hence, in how far the aforementioned four components affect our individual A’s decisions regarding compliance (cooperation) is affected by A’s attitude towards risk and further personal traits of A. From the authorities’ perspective, it appears that individual A’s level of cooperation can be affected predominantly by enforcement (action controls) and by social or moral norms (cultural controls). However, the thought experiment also suggests that the success of these controls is shaped by personal traits of individuals.

## Broadening the view: Bounded rationality and heterogeneity

Some of the assumptions of the above sketched economic perspective could be relaxed in favour of a more realistic view on individuals. In this respect, two interrelated aspects are to be mentioned. First, the ideas of individuals’ rationality are to be mentioned. With introducing bounded rationality (Simon 1959), individuals may, for example, over- or underestimate risks—e.g., the risk to get infected (component 1 above). Second, taking boundedly rational agents into consideration results in heterogeneity of agents since there are numerous forms of departing from rationality or as Axtell (2007, p. 107) puts it ``…there is one way to be rational but many ways to depart from rationality´´. It is needless to say that there is a manifold of attributes in which individuals differ and which may affect the propensity to cooperate in the pandemic management (e.g., social network, state of health, beliefs).

From the very few bits and pieces of an economically inspired model as outlined above, the relevance of personal traits of individuals regarding the effects of the policy makers’ actions becomes apparent. However, taking heterogeneity of individuals into account makes it more difficult to predict the level of voluntary cooperation of the individuals in the collective in face of a certain set of “unpleasant” actions.

## Agent-based modelling for studying cultural controls in pandemics management

Predicting the overall effects of actions in crisis management is obviously of particular relevance for policy makers. As outlined before, there is some reason to conjecture that a given set of actions may have different effects on the different individuals. However, in the pandemic crisis, policy makers have to decide on actions with respect to the macro-level, i.e., the effects emerging at the macro-level. Hence, for understanding the dynamics of a pandemic in a collective and particularly for policy advice an approach is required that allows to deal with the heterogeneity of individuals and to bridge between the individuals’ and the collective’s level.

It has been argued that agent-based modelling could be a valuable approach for predicting the effects of actions taken in a crisis situation like the Covid-19 pandemic in conjunction with human behaviour within the crisis (Adam 2020; Saltelli et al. 2020; Squazzoni et al. 2020). I agree with this view and want to stress those aspects of agent-based modelling which make it a “natural” candidate to study the pandemics management and the behavioural effects of “unpleasant” actions at the collective’s level: agent-based modelling allows to capture heterogeneous agents, puts particular emphasis on interactions among agents in a spatially and/or figuratively defined environment and seeks to understand system’s behaviour from the bottom up, i.e., agents’ behaviour (e.g., Epstein 1999). In this sense, it also allows to explicitly take policy makers as agents and their cognitive and coordinative capabilities into account (Comfort 2007) and to simulate the alternative forms of coordination for crisis management (Christensen et al. 2016).

Among the various aspects of the Covid-19 crisis that may be studied by agent-based models a particularly interesting topic could be the effects of what is captured by the term “cultural controls” referring to beliefs, norms, shared values. In this sense, during the pandemic outbreak policy makers used to emphasize, for example, mottos like “we are all in the same boat”. As illustrated in the brief thought experiment above and based on prior research, it appears that the voluntary cooperation of humans to “unpleasant” actions may be particularly shaped by (the desire of) social approval (e.g., Holländer 1990). Social approval refers to social norms which differ across cultures (Rege and Telle 2004; Twenge and Im 2007). Hence, with social norms as well as attitudes towards risk differing across cultures (Hofstede 2003), agent-based modelling may be a particularly promising approach to study the effects of pandemics management in different cultures.
